# The role of *MTHFR* C677T and *ALDH2* Glu504Lys polymorphism in acute coronary syndrome in a Hakka population in southern China

**DOI:** 10.1186/s12872-020-01410-7

**Published:** 2020-03-11

**Authors:** Jingyuan Hou, Zhixiong Zhong, Qiaoting Deng, Lifang Lin, Xing Zeng

**Affiliations:** 1 Clinical Core Laboratory, Meizhou People’s Hospital (Huangtang Hospital), Meizhou Hospital Affiliated to Sun Yat-sen University, No. 63 Huangtang Road, Meijiang District, Meizhou, 514031 People’s Republic of China; 2 Guangdong Provincial Key Laboratory of Precision Medicine and Clinical Translational Research of Hakka Population, Meizhou, 514031 People’s Republic of China; 3 Guangdong Provincial Engineering and Technological Research Center for Molecular Diagnostics of Cardiovascular Diseases, Meizhou, 514031 People’s Republic of China; 4 Center for Cardiovascular Diseases, Meizhou People’s Hospital (Huangtang Hospital), Meizhou Hospital Affiliated to Sun Yat-sen University, Meizhou, 514031 People’s Republic of China

**Keywords:** Acute coronary syndrome, Methylenetetrahydrofolate reductase, Aldehyde dehydrogenase 2, Gene polymorphism, Hakka

## Abstract

**Background:**

Acute coronary syndrome (ACS) is the most serious type of coronary heart disease and is a global medical burden. The pathogenesis of ACS is very complex and still poorly understood. Epidemiologic studies have revealed that the manifestation of ACS are the results of the interactions between multiple environmental and genetic factors. The present study aimed to investigate the role of polymorphisms of *MTHFR* C677T and *ALDH2* Glu504Lys as risk factors for ACS in a Hakka population in southern China.

**Methods:**

Between September 1, 2015 and October 31, 2017, a total of 1957 individuals, including 860 ACS patients and 1097 controls were recruited. Blood samples were collected and genotypes were determined by DNA microarray chip method and direct sequencing method.

**Results:**

For the *MTHFR* C677T polymorphism, frequencies of *CC*, *CT*, and *TT* genotypes were 53.60% versus 55.33, 39.53% versus 38.65 and 6.86% versus 6.02% in patients with ACS versus controls, respectively(*p* > 0.05). The differences in genotype frequencies between the ACS patients and controls in the three genetic model were not statistically significant. For the *ALDH2* Glu504Lys polymorphism, the frequencies of ALDH2*1*1, ALDH2*1*2, and ALDH2*2*2 genotypes were 48.72, 42.67 and 8.6% in the ACS patients, respectively, while these were 53.33, 39.11 and 7.57% in the controls, respectively, showing no significant difference in the distribution of the *ALDH2* genotype between the groups. Using the wild genotype ALDH2*1*1 as reference, relative risk analysis revealed a slightly increased risk for ACS in individuals with the ALDH2*1*2 plus ALDH2*2*2 genotypes (odds ratio (OR) = 1.203, 95% confidence interval (CI) = 1.006–1.438, *p* = 0.043). In a multivariate logistic regression model, even after adjusting for potential covariates, the association between ALDH2 *2 allele and ACS remained significant (OR = 1.242, 95% CI = 1.045–1.561, *p* = 0.038).

**Conclusions:**

We present findings regarding the possible clinical impact of the ALDH2*2 variant on ACS patients in a Hakka population in southern China and our findings might help to stratify the high-risk ACS patients and implement appropriate strategies for this genetic subpopulation to ultimately guide the precision preventive procedures in the future.

## Background

Acute coronary syndrome (ACS) is the most serious type of coronary heart disease and is a global medical burden [1]. ACS refers to a broad spectrum of clinically manifest coronary artery disease compatible with unstable angina and myocardial infarction (MI) with or without ST elevation [2–4]. Indeed, the pathophysiology, clinical presentation and prognosis of ACS are extremely heterogeneous [5, 6]. Patients identified as high-risk groups may be undertook earlier health management and more aggressive treatment, and thus early identifying individual at risk of ACS is of paramount importance [7, 8]. ACS is a complex disorder that involves multiple environmental and genetic factors. It has been generally reported that lifestyle such as low physical activity, obesity, smoking, drinking and fast food intake are found to be associated with patients with ACS [9–11]. However, since the traditional environment and lifestyle factors reflects only a small part of mechanisms related to the development of ACS, whereas the relationship between genetic molecular mechanisms and future ACS, was not addressed [12, 13].

Methylenetetrahydro folate reductase (*MTHFR*) plays a significant role in cellular metabolism of folate, as well as in the synthesis of purine, DNA, and RNA and is essential in the methyl cycle that converts homocysteine (Hcy) to methionine [14, 15]. *MTHFR* is a crucial controlling enzyme related to Hcy metabolism and many articles connecting *MTHFR* single nucleotide polymorphism (SNP), mostly *MTHFR* C677T, with plasma Hcy levels [16]. There are growing evidences that elevated plasma Hcy concentration confers an increased risk for cerebrovascular, peripheral vascular disease and the occurrence of cardiovascular disease in patients with or without hypertension, diabetes mellitus and other conventional risk factors [17–19]. However, to date, most studies of *MTHFR* C677T polymorphism on ACS are not fully understood.

Mitochondrial aldehyde dehydrogenase 2 (*ALDH2*) has been proved to responsible for oxidation and detoxification of aromatic and aliphatic aldehydes [20]. The significant functional polymorphism of the *ALDH2* gene is at exon 12, wherein glutamate acid (Glu) is replaced by lysine (Lys) at position 504(Glu504Lys) [21]. Carriers of the variant *ALDH2* allele (ALDH2*2) have dramatically deficient enzymatic activity by approximately 60 to 80% in heterozygotes and found in approximately 560 million people of East Asian descent but are virtually non-existent in other populations of the world [22, 23]. Numerous studies suggest that variations of *ALDH2* are associated with the risk for a series of diseases such as alcoholic cirrhosis, cancer, Alzheimer’s and stroke [24–26]. Moreover, the reduced activity of the mitochondrial *ALDH2* causes accumulation of acetaldehyde and 4-hydroxy-2-nonenal (4-HNE) and decrease the anti-oxidative stress effects, which has been proposed to be potentially linked with coronary artery disease (CAD) [27–29].

To the best of our knowledge, there is currently no report that evaluated the role of polymorphisms of *MTHFR* C677T and *ALDH2* Glu504Lys in ACS patients in a Hakka population in southern China. We hypothesized that polymorphisms of the *ALDH2* and *MTHFR* gene that result in reduced enzyme activity may modify the risk of ACS. We tested this hypothesis in a case-control study.

## Methods

### Study population

This was a retrospective study that analyzed consecutive patients who visited the cardiovascular medicine of our hospital, which is a tertiary hospital located in urban area between September 1, 2015 and October 31, 2017. The annual patients admitted to the cardiovascular medicine were ranged from 10,000 to 12,000 patients. Participants of this study include native inhabitants in Meizhou region for at least 3 generations with no migration history, from both rural and urban origins. A total of 1957 individuals, including 860 ACS patients and 1097 controls were recruited. ACS patients were diagnosed by two senior specialists at the time of the index hospitalisation according to the same clinical criteria defined by the European Society of Cardiology/American College of Cardiology. Control subjects were free of cardiac disorders according to medical history, physical and laboratory examinations. The present study complies with the Declaration of Helsinki and was approved by the ethics committee of the Meizhou People’s Hospital (Huangtang Hospital), Meizhou Hospital Affiliated to Sun Yat-sen University. A prior written informed consent was obtained from patients and controls who participated in the present study before inclusion.

### Definitions

The ACS was classified as ST segment elevation myocardial infarction (STEMI), non-ST segment elevation myocardial infarction (NSTEMI) and unstable angina pectoris (UA) based on clinical, electrocardiographic, and biochemical criteria. In brief, STEMI was defined as presence of persistent chest pain along with at least one of the following: new-onset ST segment elevation ≥1 mm in at least 2 contiguous leads in the admission electrocardiogram, or left bundle branch block and elevated cardiac biomarker values above the upper limit of normal range. NSTEMI was defined as presence of persistent chest pain, absence of new-onset ST segment elevation, and/or the presence of T-wave abnormalities and elevated cardiac biomarker above the upper limit of normal range. UA was defined as worsening angina without elevated values of cardiac biomarker. Patients were excluded if they had a previous history of ACS, percutaneous coronary intervention or coronary artery bypass graft surgery, malignant tumor, blood system diseases, autoimmune diseases and severe hepatic or renal dysfunction.

Information on the age, gender, smoker, and drinker, hypertension, diabetes and dyslipidemia were collected from the medical records. Smoker was defined as those daily smoking for more than 5 cigarettes. Drinker was defined as a person who drinks hard liquors at least once per week and continuously drinks for more than a year. Hypertension was defined as history of hypertension or systolic blood pressure (SBP) ≥ 140 mmHg or diastolic BP (DBP) ≥ 90 mmHg. Diabetes was defined as history of diabetes or a fasting plasma glucose level of ≥7.0 mmol/L or 2-h OGTT venous blood glucose level ≥ 11.1 mmol/L and a hemoglobin A1c level of ≥6.5%. Dyslipidemia was defined as level of triglycerides (TG) value of ≥1.50 mmol/L or LDL cholesterol (LDL-c) value of ≥3.92 mmol/L or under medication of lipid lowering drugs.

### Blood sampling and genotyping

Five millilitres of venous blood was collected in ethylene diamine tetra-acetic acid (EDTA) tubes from each participant. Genomic DNA extraction was carried out with the use of a DNA extraction kit (Qiagen, Hilden, Germany) following the manufacturer’s instructions and quantified using Nanodrop 2000 Spectrophotometer (Thermo Scientific, Wilmington, DE, USA). The extracted DNA was dissolved in sterile distilled water and stored at − 20 ͦC before used. *ALDH2* Glu504Lys and *MTHFR* C677T genotyping was performed used the DNA microarray chip method and direct sequencing method as previously described [23]. To verify our results, genotyping was randomly repeated in 10% of the patients with the sanger sequencing method. All the repeated experiments revealed identical results when compared with the initial genotyping.

### Statistical analysis

Data were collected and all statistical analyses were performed with SPSS 19.0 (SPSS Inc., Chicago, IL, USA). Continuous variables, which were expressed as mean ± standard deviation (SD) and analysed by t test or Mann-Whitney U test. The categorical variables were expressed as percentage and analysed by Chi-square test or Fisher’s exact test. Hardy-Weinberg equilibrium (HWE) was used to assess the representativeness of the participants. Univariable logistic regression analysis was used to test the association between ACS and gene polymorphisms. To examine the association between ACS and possible confounding factors, univariate and multivariate logistic regression analysis were performed. The correlation risk was estimated by odds ratio (OR) and 95% confidence interval (95% CI). A *p* value of < 0.05 was considered statistically significant.

## Results

### The demographic and clinical characteristics of the study population

In this study, we analysed 860 ACS patients and 1097 control subjects. The demographic and baseline clinical characteristics of the study population were shown in Table 1. The proportion of male, smokers, hypertension, diabetes and dyslipidemia cases were higher than those of controls and showed statistical significance (*p* < 0.05). Besides, ACS patients had elder age, higher SBP and DBP, higher serum total cholesterol (TC), LDL-c and TG levels than control subjects (*p* < 0.05). No significant differences were found with regards to the proportion of drinkers, serum high-density lipoprotein cholesterol (HDL-c), Apolipoprotein A and Apolipoprotein B levels between the two groups (all *p* > 0.05).

**Table 1:** The demographic and clinical characteristics of the study population

Characteristics	ACS	Controls	*p-*value
	(*n* = 860)	(*n* = 1097)	
Age (year)	67.6 ± 11.5	65.7 ± 11.7	< 0.001
Male (n, %)	633 (73.6)	709 (64.6)	< 0.001
SBP (mmHg)	133.3 ± 25.7	126.1 ± 18.7	< 0.001
DBP (mmHg)	82.6 ± 14.91	79.0 ± 14.8	< 0.001
Smokers (n, %)	230 (26.7)	166 (15.1)	< 0.001
Drinkers (n, %)	113 (13.1)	124 (11.3)	0.217
Hypertension, n(%)	421 (49.0)	442 (40.3)	< 0.001
Diabetes, n(%)	281 (32.7)	219 (20.0)	< 0.001
Dyslipidemia, n(%)	266 (30.9)	277 (25.3)	0.005
TG (mmol/L)	1.74 ± 0.80	1.92 ± 0.83	0.036
TC (mmol/L)	5.06 ± 1.35	4.76 ± 1.45	< 0.001
HDL-c (mmol/L)	1.25 ± 0.33	1.26 ± 0.41	0.736
LDL-c (mmol/L)	2.90 ± 0.98	2.68 ± 1.00	< 0.001
APOA (mmol/L)	1.10 ± 0.27	1.08 ± 0.35	0.298
APOB (mmol/L)	0.86 ± 0.29	0.85 ± 0.29	0.974

*SBP* Systolic blood pressure, *DBP* Diastolic blood pressure, *TG* Triglyceride, *TC* Total cholesterol, *HDL-c* High-density lipoprotein cholesterol, *LDL-c* low-density lipoprotein cholesterol, *APOA* Apolipoprotein A, *APOB* Apolipoprotein B

### Genotype and allele distributions in ACS patients and controls

Table 2 shows the allele and genotype frequencies of *MTHFR* C677T and *ALDH2* Glu504Lys polymorphisms in ACS patients and controls. The genotype distributions of the two SNPs in the ACS patients (χ^2^ = 0.119, *p* = 0.730 and χ^2^ = 0.254, *p* = 0.614, respectively) and controls (χ^2^ = 0.504, *p* = 0.478 and χ^2^ = 0.126, *p* = 0.723, respectively) were consistent with the HWE(*p* > 0.05). The effect of the two SNPs on the risk of ACS was also performed based on dominant (normal homozygote vs. heterozygote plus mutant homozygote), recessive (normal homozygote plus heterozygote vs. mutant homozygote) and additive (normal vs. heterozygote or vs. mutant homozygote) models of inheritance. For the *MTHFR* C677T polymorphism, frequencie*s* of CC, CT, and TT genotypes were 53.60% versus 55.33, 39.53% versus 38.65 and 6.86% versus 6.02% in patients with ACS versus controls, respectively(*p* > 0.05). The differences in genotype frequencies between the ACS and control groups in the three genetic model were not statistically significant. Another, the T allele frequency difference between the two groups was also not statistically significant (*p* = 0.362).

**Table 2:** Genotype and allele distributions in ACS patients and controls

SNP	Model	Genotype	ACS(n,%)	Controls(n,%)	OR	95% CI	*p*-value
*MTHFR* C677T	Dominant	CC	461 (53.60)	607 (55.33)	1.000	reference	
		CT + TT	399 (46.40)	490 (44.67)	1.072	0.896–1.283	0.446
	Recessive	CC + CT	801 (93.14)	1031 (93.98)	1.000	reference	
		TT	59 (6.86)	66 (6.02)	1.151	0.800–1.654	0.449
	Additive	CC	461 (53.60)	607 (55.33)	1.000	reference	
		CT	340 (39.53)	424 (38.65)	1.056	0.876–1.273	0.569
		TT	59 (6.86)	66 (6.02)	1.177	0.812–1.707	0.389
		C allele	1262 (73.37)	1638 (74.66)	1.000	reference	
		T allele	458 (26.63)	556 (25.34)	1.069	0.926–1.235	0.362
		HWE	χ^2^ = 0.119, *p* = 0.730	χ^2^ = 0.504, *p* = 0.478			
*ALDH2* Glu504Lys	Dominant	*1*1	419 (48.72)	585 (53.33)	1.000	reference	
		*1*2 + *2*2	441 (51.28)	512 (46.67)	1.203	1.006–1.438	0.043
	Recessive	*1*1 + *1*2	786 (91.40)	1014 (94.43)	1.000	reference	
		*2*2	74 (8.60)	83 (7.57)	1.150	0.829–1.595	0.401
	Additive	*1*1	419 (48.72)	585 (53.33)	1.000	reference	
		*1*2	367 (42.67)	429 (39.11)	1.194	0.990–1.441	0.063
		*2*2	74 (8.60)	83 (7.57)	1.245	0.888–1.745	0.203
		*1 allele	1205 (70.06)	1599 (72.88)	1.000	reference	
		*2 allele	515 (29.94)	595 (27.12)	1.149	0.999–1.321	0.052
		HWE	χ^2^ = 0.254, *p* = 0.614	χ^2^ = 0.126, *p* = 0.723			

*SNP* Single nucleotide polymorphism, *OR* Odds ratio, *CI* Confidence interval, *HWE* Hardy-Weinberg equilibrium

For the *ALDH2* Glu504Lys polymorphism, the frequencies of ALDH2*1*1, ALDH2*1*2, and ALDH2*2*2 genotypes were 48.72, 42.67 and 8.6% in the ACS patients, respectively, while these were 53.33, 39.11 and 7.57% in the controls, respectively, showing no significant difference in the distribution of the *ALDH2* genotype between the groups. We further analysed this association in dominant model (normal homozygote ALDH2*1*1 vs. heterozygote ALDH2*1*2 plus mutant homozygote ALDH2*2*2). Using the wild genotype ALDH2*1*1 as reference, relative risk analysis revealed a slightly increased risk for ACS in individuals with the ALDH2*1*2 plus ALDH2*2*2 genotypes (OR = 1.203, 95% CI = 1.006–1.438, *p* = 0.043). However, the differences in genotype frequencies between the ACS patients and control groups in the recessive and additive model were not statistically significant. Meanwhile, there was no significant difference between the ACS patients and control groups in the ALDH2*2 allele frequencies (*p* = 0.052).

### Risk factors of ACS patients by univariate and multivariate regression

We performed multivariate logistic regression analysis to identify the variables that independently and significantly contributed to the presence of ACS (Table 3). Multivariate logistic regression analysis after adjustment for other established risk factors: age, gender, smoking status, drinking status, hypertension, diabetes and dyslipidemia, showed that carrier of ALDH2 *2 allele was an independent risk factor for ACS (OR = 1.242, 95% CI = 1.045–1.561, *p* = 0.038). As regards other covariates, age, gender, smoking status, hypertension, diabetes and dyslipidemia were independent risk factors for ACS (all *p* < 0.05). By contrast, drinking status and carrier of T allele in *MTHFR*, exerted no significant effect on ACS (all *p* > 0.05).

**Table 3:** Univariate and multivariate logistic regression analysis for risk factors in ACS patients

Variable	Univariate			Multivariate		
	OR	95%CI	*p*-value	OR	95%CI	*p*-value
Age	1.362	1.137–1.631	0.001	1.514	1.250–1.835	< 0.001
Gender	1.526	1.255–1.856	< 0.001	1.385	1.118–1.716	0.003
Smoking	2.048	1.637–2.561	< 0.001	2.284	1.762–2.961	< 0.001
Drinking	0.751	0.477–1.183	0.217	0.831	0.513–1.274	0.229
Hypertension	1.421	1.187–1.702	< 0.001	1.387	1.147–1.676	0.001
Diabetes	1.946	1.584–2.390	< 0.001	1.939	1.567–2.398	< 0.001
Dyslipidemia	1.326	1.087–1.617	0.005	1.317	1.069–1.622	0.010
*MTHFR T* allele carriers	1.072	0.896–1.283	0.446	1.106	0.933–1.358	0.289
ALDH2 *2 allele carriers	1.203	1.006–1.438	0.043	1.242	1.045–1.561	0.038

*OR* Odds ratio, *CI* Confidence interval

## Discussion

ACS is an important global public health issue and one of the leading causes of mortality rate, as well as a great economic burden for society worldwide [1]. The pathogenesis of ACS is very complex and still poorly understood. Epidemiological evidences have revealed that the manifestation of ACS are the results of the interactions between genetic and environmental risk factors [10, 12]. Discovery of genetic risk factors is of great interest in clinical practice, and will help improve the prevention of ACS and enact effective health care policies. In the present study, we for the first time investigated the association of *MTHFR* C677T polymorphism and *ALDH2* Glu504Lys polymorphism with ACS in a Chinese Hakka population. Next we performed genotype association test with dominant, recessive, and additive models. Our results indicated that ACS patients with the mutant *ALDH2* (dominant model) had an increased risk of ACS (OR = 1.203, 95% CI = 1.006–1.438, *p* = 0.043). In a multivariate logistic regression model, even after adjusting for potential covariates, the association between ALDH2 *2 allele and ACS remained significant (OR = 1.242, 95% CI = 1.045–1.561, *p* = 0.038).

*MTHFR* is one of the most important enzymes with a pivotal role in the Hcy metabolism [14]. Accumulating evidence suggests that there was a strong link between the *MTHFR* gene C677T polymorphism and plasma Hcy levels [15]. Consequently, the relatively common C677T mutation in *MTHFR* might be an important genetic risk factor for ischemic heart disease through its effects on Hcy metabolism [16, 18]. The implication of the *MTHFR* gene in CAD pathogenesis has been extensively studied in several ethnic groups while the results were inconsistent. A meta-analysis of the risk of CAD related to the *MTHFR* C677T polymorphism showed that individuals with TT genotype have a significantly higher risk of CAD [30]. The study of Nakai et al. in a Japanese population has shown that the incidence of TT mutation in acute angina patients is significantly higher than the healthy population [31]. Helfenstein et al. revealed that the *MTHFR* TT genotype and allele frequencies in patients with MI are higher than those in healthy individuals [32]. Another meta-analysis of case-control studies found that *MTHFR* C677T polymorphism was associated with risk of MI in young and middle-aged populations, particularly among Caucasians [33]. However, there were some conflicting results. Contrary to previous literatures, our results in the present study did not showed such a positive association between the *MTHFR* C677T polymorphism and elevated risk of ACS. Similar findings were also observed in ACS patients of Asian, Caucasian and African descent [34–36]. The discrepancy between the results from across several studies is unclear and may be due to differences in nutritional intake or deficiencies required for the *MTHFR* pathway, such as folate, pyridoxal phosphate (B6) or methylcobalamin (B12), or ethnic populations and genetic backgrounds exist [37–39].

ALDH2 is the major enzyme that eliminates exogenous and endogenous toxic aldehydes, such as acetaldehyde, 4-HNE and environmental aldehydes like acrolein [40]. Up to 40% of East Asians carry a variant *ALDH2* with deficient catalytic activity [22]. Importantly, recent studies indicate that carriers of ALDH2*2 mutation have an increased risk for alcoholic cirrhosis, cancer and CAD [25, 27]. Prior studies has provided evidences that *ALDH2* protects against myocardial ischemia/reperfusion (I/R) injury by removing toxic aldehydes such as 4-HNE in animal models of myocardial ischemia [28, 41]. Jo et al. suggested that genotypes carrying the mutant *ALDH2* allele were significantly more frequent in patients with MI than in the controls, indicating that *ALDH2* gene is a risk factor for MI [42]. A meta-analysis by Gu et al. provides strong evidence that *ALDH2* Glu504Lys polymorphism may be associated with increased risk of CAD and MI in East Asians, especially among Chinese and Korean populations [21, 43]. Interestingly, in the present study, a similar result was revealed that *ALDH2* mutant genotypes (*1/*2 plus *2/*2) might serve as an independent genetic risk factors for ACS in a Hakka population in southern China, which was consisted with a previous study in Han Chinese [44]. The underlying mechanism between *ALDH2* Glu504Lys polymorphism and risk of ACS is considered multifactorial. The genetic variation in the *ALDH2* gene can influence serum high-density lipoprotein-C levels and intracellular asymmetric dimethylarginine levels, subsequently increase the risk of ACS [21, 45]. The *ALDH2* genetic polymorphism was also significantly associated with alcohol consumption and increase blood pressure, and others has revealed a beneficial role of *ALDH2* against alcohol, acetaldehyde and toxic aldehyde-induced reactive oxygen species formation, and tissue injury [21, 46–48]. However, the exact mechanism still needs further verification.

It should be noted that the results of this observational study have some limitations. Firstly, the main study limitation was based upon the retrospective design of the study and patient selection biases could not be avoided. Secondly, this study is a single-center study and the sample size of the study may not be sufficiently large. To further validate the association between ALDH2*2 variant and the risk of ACS patients, additional studies employing larger cohorts will be needed in the future.

## Conclusion

The present study showed the possible clinical impact of the ALDH2*2 variant on ACS patients in a Hakka population in southern China. We hope our findings might help to stratify the high-risk ACS patients and implement appropriate strategies for this genetic subpopulation to ultimately guide the precision preventive procedures. However, more efforts is urgently required to confirm this hypothesis in the future.

## Data Availability

The datasets generated during the current study are available from the corresponding author on reasonable request.

## Abbreviations

4-HNE: 4-hydroxy-2-nonenal
ACS: Acute coronary syndrome
ALDH2: Aldehyde dehydrogenase 2
APOA: Apolipoprotein A
APOB: Apolipoprotein B
CAD: Coronary artery disease
CI: Confidence interval
DBP: Diastolic blood pressure
EDTA: Ethylene diamine tetra-acetic acid
Hcy: Homocysteine
HDL-c: High-density lipoprotein cholesterol
HWE: Hardy-Weinberg equilibrium
LDL-c: Low-density lipoprotein cholesterol
MI: Myocardial infarction
MTHFR: Methylenetetrahydro folate reductase
NSTEMI: Non-ST segment elevation myocardial infarction
OR: Odds ratio
SBP: Systolic blood pressure
SD: Standard deviation
SNP: Single nucleotide polymorphism
STEMI: ST segment elevation myocardial infarction
TC: Total cholesterol
TG: Triglycerides
UA: Unstable angina
